# Macrocycles in Bioinspired Catalysis: From Molecules to Materials

**DOI:** 10.3389/fchem.2021.635315

**Published:** 2021-03-26

**Authors:** Jie Shang, Yao Liu, Tiezheng Pan

**Affiliations:** ^1^ School of Life Sciences, Northwestern Polytechnical University, Xi’an, China; ^2^ State Key Laboratory of Analytical Chemistry for Life Sciences, School of Chemistry and Chemical Engineering, Nanjing University, Nanjing, China

**Keywords:** macrocycle, catalysis, cyclodextrin, cucurbituril, rotaxane, porphyrin

## Abstract

Macrocyclic compounds have been studied extensively as the host molecules in supramolecular chemistry. Their structural characteristics make macrocycles desirable in the field of molecular recognition, which is the key to high catalytic efficiencies of natural enzymes. Therefore, macrocycles are ideal building blocks for the design of bioinspired catalysts. This mini review highlights recent advances ranging from single-molecule to metal-organic framework materials, exhibiting multilevel macrocycle catalysts with unique catalytic centers and substrate-binding affinities.

## Introduction

Natural enzymes are innately endowed with substrate-binding sites, which are essential for the formation of enzyme-substrate complex and crossing the activation energy barrier of catalytic reactions. These binding sites are generated by the elaborated folding of peptides into cavity-like conformations that are spatially complementary to the substrates. From the perspective of supramolecular chemistry, many synthetic macrocycles provide the structural feature of substate-binding sites, which leads to a broad application of macrocyclic compounds in the areas of ion recognition (Ding et al., 2017), gas storage (Zhou et al., 2020), stimuli-responsive materials (Lei et al., 2020), drug delivery (Webber and Langer, 2017), and catalysis (Pairault et al., 2020). Most of all, the nanosized confined spaces in the macrocycles are excellent platforms for highly efficient and selective catalytic reactions. With the development of host-guest chemistry, cyclodextrins (Breslow, 1982), calixarenes (Li et al., 2020a; Li et al., 2020b), pillararenes (Xiao et al., 2018; Xiao et al., 2019), cucurbiturils (Wagner et al., 2020), and many other macrocyclic compounds with enzyme-like substrate affinity and selectivity, have been studied for specific molecular recognition, binding, and bioinspired catalysis. The rising hotspot of porphyrins-based polymers and crystalline organic materials has produced tremendous achievements with high guest uptake performance and structural stability (Farha et al., 2011). Inspired by the natural enzymes, macrocycle-based systems, including single molecules (Dong et al., 2012), supramolecular macrocycle systems (Lewandowski et al., 2013), covalently linked porphyrins (Cheung et al., 2019), and metal-organic framework materials (Shultz et al., 2009), become an indispensable role as bioinspired catalysts.

Except for the cavity-like structure, the convenient modification of macrocycles provides unlimited possibilities to mimic the active sites of natural enzymes. The functionalization of supramolecular macrocyclic structures brought dynamic control of the activity and enantioselectivity into organic catalysis. For instance, the selenium-incorporated cyclodextrins (CDs) were used as a glutathione peroxidase mimic for their antioxidant activity (Huang et al., 2011). Moreover, macrocycles functionalized with transition metals showed specific activity for asymmetric catalytic reactions (Li et al., 2015a; Li et al., 2015b). Various catalytic groups have expanded the application scope of macrocycles in both enzyme catalysis and organocatalysis. Also, the supramolecular assembly and covalent crosslinking of macrocycles further take advantage of the cluster effect to enhance their structural stability, material performance, and catalytic activity. This review covers the recent work of macrocycle-based catalysts ranging from the synthetic macrocycles, self-assembled supramolecular scaffolds, to nanoscale materials. These works have facilitated both the academic study and industrial applications of macrocyclic catalysts.

## Single-Molecule Macrocycles as Bioinspired Catalysts

**FIGURE 1:**
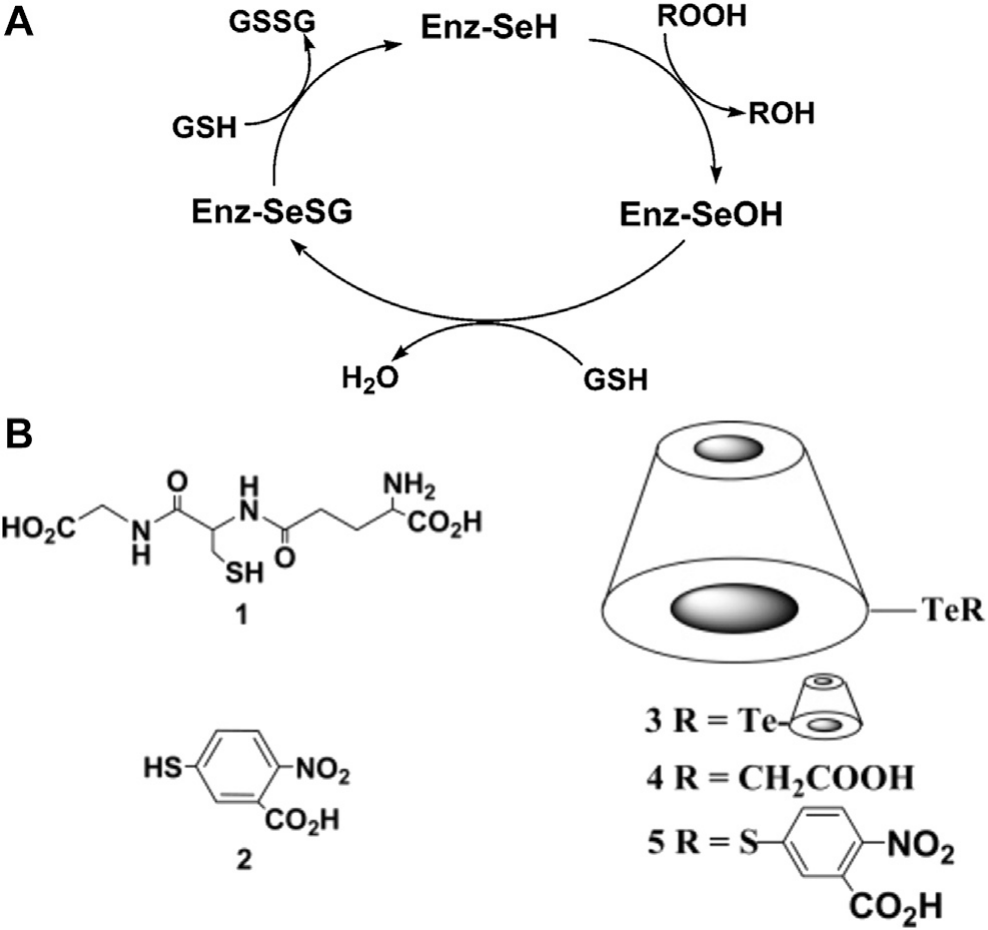
**(A)** The catalytic mechanism of GPx. GPxs catalyze the reduction of peroxides at the expense of glutathione molecules, involving the formation and breaking of Se-S covalent bond. **(B)** Chemical structures of the substrates of GPx (left) and the tellurium-containing CD (right). A and B Reproduced from Dong et al. (2004) with permission. Copyright 2004 American Chemical Society.

Supramolecular chemistry has brought a great opportunity to the development of macrocycle catalysis. To mimic the cavity of natural enzymes, many single-molecule macrocycles, including CDs, cyclophanes, cavitands, calixarenes have been developed (Ikeda and Shinkai, 1997; Diederich et al., 2008; Brotin and Dutasta, 2009; Hooley and Rebek, 2009; Klöck et al., 2009; Schühle et al., 2011). These macrocycles could promote the reactions in a confined hydrophobic space and carry the reactions in a specific path in a bioinspired manner with specificity and selectivity. Following the pioneering work contributed by Breslow (Breslow, 1982), Tabushi (Tabushi, 1982), Saenger (Saenger, 1980), and D’Souza and Bender (D’Souza and Bender, 1987), many CD-based artificial enzymes have been extensively developed. In addition, CD derivatives are widely used as scaffolds for the construction of artificial glutathione peroxidase (GPx), an efficient antioxidant enzyme with the selenium catalytic center (Figure 1A). Numerous CD-based GPx models have been designed via incorporating selenium/tellurium functional groups on macrocyclic building blocks (Huang et al., 2011). By modification of CD and with ditelluride moiety, 2,2′-ditellurobis (2-deoxy-β-CD) showed excellent GPx-like activity in the presence of substrate thiols (Figure 1B) (Liu et al., 1998; Liu et al., 2000; Liu et al., 2002; Dong et al., 2004; McNaughton et al., 2004; Dong et al., 2006a; Dong et al., 2006b; Dong et al., 2007). When an aromatic substrate was used instead of natural substrate glutathione, the activity could be 200,000-fold more efficiently than the common GPx mimic PhSeSePh (Dong et al., 2004). The kinetic constant was similar to that of natural GPx (10^7^ M^−1^min^−1^). These single-molecule macrocyclic catalysts broke the traditional design strategy focusing on organocatalytic mechanisms, paying more attention to the molecular binding affinity to the substrate.

**FIGURE 2:**
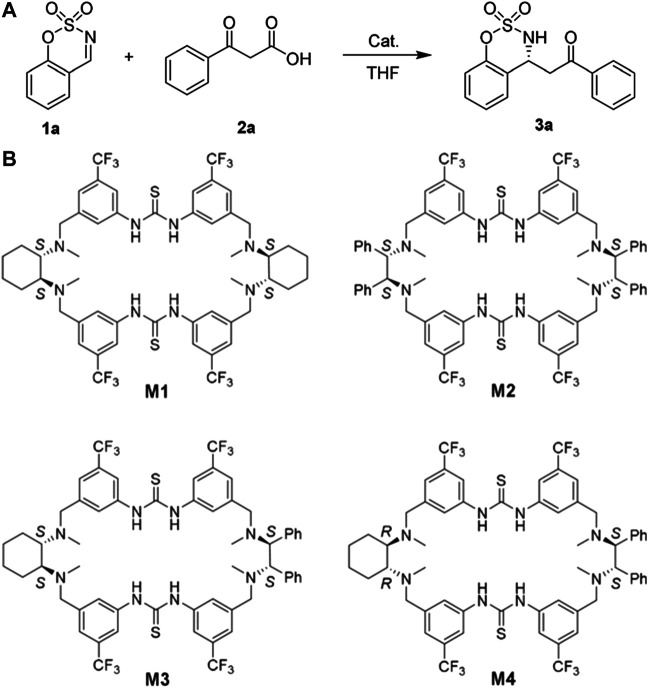
**(A)** The decarboxylative Mannich reaction ‘promoted by chiral macrocycle catalysts for asymmetric catalysis. **(B)** The effects of different macrocyclic conformations and chiral microenvironments on catalytic efficiency and enantioselectivity were studied by evaluating the catalytic behaviors of macrocycle M1-M4. M4 achieved a 99% yield and 94:6 enantiomeric ratio (er) under optimal conditions, while M1-M3 gave a relatively lower. A and B Reproduced from Guo et al. (2020) with permission. Copyright 2020 Wiley-VCH Verlag GmbH & Co. KGaA, Weinheim.

Besides CDs, more kinds of macrocyclic compounds have been developed to push the boundary of macrocycle catalysis to heterogeneous catalysis and asymmetric catalysis. Reisner, Scherman, and coworkers presented the surface-adsorbed host-guest interactions (Wagner et al., 2020). In this work, the reduction of CO_2_ to CO was highly corelated to the complexation behaviors of cucurbit (6)uril [CB (6)]. It was proved that the reaction site located inside the cavity of CB (6) just like the binding between enzymes and their substrates. The experiment results indicated that differences between this CB (6)-based electrocatalytic reaction and traditional CO_2_ reduction existed, but not reflected in the H_2_ production of the reaction process. Therefore the unique CO_2_-hosting ability of CB (6) played the critical role in the catalysis. Also, an efficient enantioselective reaction was reported by Wang’s group, showing that chiral macrocyclic compounds with delicate hydrogen-bonding network could conduct an artificial dimerization (Guo et al., 2020). The catalytic cavity based on the macrocyclic compounds was achieved by dimerization for the catalytic reaction (Figure 2). Inside the catalytic cavity, the Mannich reaction of cyclic aldimine substrates was promoted by this assembly system. The imine substrate was activated by the H-bond network that generated through the dimerization. Besides, Wang’s group developed a counteranion trapping strategy with macrocyclic compounds for enantioselective catalysis. The macrocycles contained cooperative moieties to construct a chiral catalytic cavity (Ning et al., 2020). Their catalytic microenvironment was further optimized with functionalization for the regulation of substrate-binding. These catalytic macrocycles exhibited high yield and stereoselectivity in the Friedel-Crafts reaction using ethanedisulfonic acid as substrates. The high activity was attributed to the catalytic cavity of macrocycles that enhanced the acidity and ion-pairing. These works above showed that the different properties of macrocyclic compounds, such as hosting ability, cavity size, structural symmetry, and electrostatic distribution, could largely enrich the variety of potential catalytic reactions for macrocyclic catalysts.

## Supramolecular Macrocycle Systems as Bioinspired Catalysts

Compare to single-molecule macrocycles, supramolecular macrocycle systems consist of not only one macrocycle, but also other components that non-covalently bound to the host molecule (e.g., the axle and guest molecules), allowing multiple catalytic behaviors and functionalization possibilities. Leigh’s group developed a rotaxane structure that showed ribosome-like activity to synthesize peptides in a successive manner. (Lewandowski et al., 2013). The substrate amino acids were linked sequentially to a strand, and a tethered thiol-functionalized macrocycle transported the amino acids on to another end of the strand to form a new peptide oligomer. The catalytic system represents a significant step to mimic the function of ribosomes chemically. The dynamic feature of supramolecular structures provides “smart control” over the catalytic reaction. Inspired by the rotaxane catalysis and the trigger-induced effects that regulate enzymatic syntheses, a pH-responsive pseudorotaxane switch was constructed with a CB (6) macrocycle and an organoselenium molecular strand (Li et al., 2015). The organoselenium strand contains an antioxidant selenium center for catalysis and two regulation imino groups to position the CB (6) macrocycle along the strand. When pH < 6, no enzyme activity was observed as the active site was concealed by CB (6). When pH > 7, the active site was exposed as CB (6) bound to the imino groups. Therefore, the enzyme activity of the complex was turned on. Although these supramolecular macrocycle systems do not focus on the substrate binding ability, they could mimic the activity-regulation mechanism of natural enzymes to control the catalytic reaction process.

**FIGURE 3:**
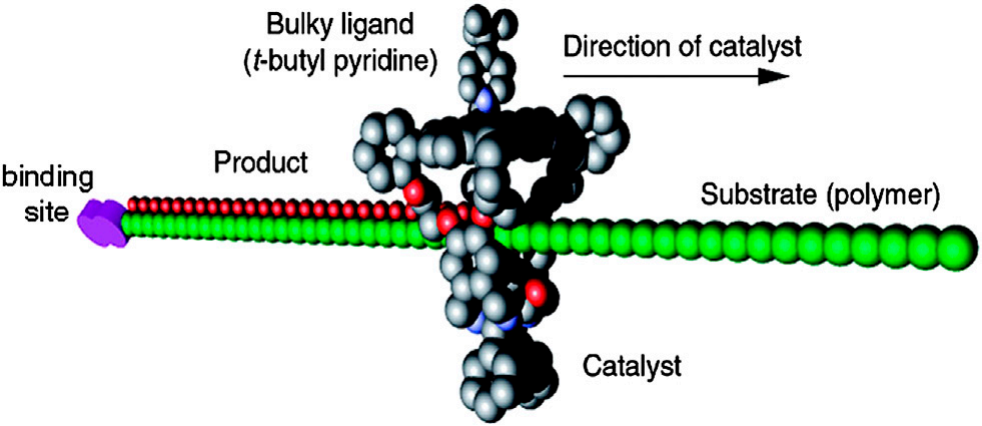
The processive catalytic system containing a linear polymer and a macrocycle. The macrocycle is modified with catalytic center and restrained to the polymer, which acts as axle molecule in the rotaxane system. As the macrocycle moves from one end to the other, the axle is functionalized (e.g., oxidation) sequentially by the catalyst. Reproduced from Deutman et al. (2014) with permission. Copyright 2014 American Chemical Society.

The supramolecular macrocycle systems were also applied in the study of processive catalysis (Figure 3). Inspired by the DNA polymerase that operates by allowing multiple catalysis rounds to occur, artificial catalytic supramolecular structures acting in this processive manner were constructed. A porphyrin-containing rotaxane system with a manganese macrocycle was developed for polybutadiene epoxidation (Thordarson et al., 2003; Coumans et al., 2006; Monnereau et al., 2010; Deutman et al., 2014). The macrocycle could act as a catalytic site and catalyze polybutadiene into 80% *trans*- and 20% *cis*-epoxide polybutadieneepoxide. This stereoselectivity difference indicated the steric mechanism of the reaction. Takata’s group synthesized a macrocyclic rotaxane containing Pd moiety for hydroamination (Miyagawa et al., 2010). The processive catalysis took place inside the cavity of the macrocycle, like enzyme catalysis. Also, Harada’s group introduced a molecular clamp that polymerized δ-valerolactone (δ-VL) (Takashima et al., 2011). It consists of *a*- and *ß*-CD dimer connected with terephthalamide. The system afforded poly (δ-VL) with a number-average molecular Weight (Mn) = 11,000 and that a mixture of *a*- and *ß*-CDs without dimerization afforded Mn = 2,300. The processive catalysis was hardly achieved by traditional catalysts, and it also requires plenty of protein components and complicated mechanism in nature. However, this special catalytic behavior based on supramolecular macrocycle systems could play a critical role in the modification of polymers, peptides, and other macromolecules in future.

## Covalently Linked Porphyrins as Bioinspired Catalysts

Porphyrin is a heteroatom-containing macrocyclic compound constructed by four pyrrole units which are connected by four methines as the bridge and are in a planar conformation to form 18π electrons conjugation system, and porphyrin can coordinate with many metals to form metallated porphyrins with extensive applications in many scientific fields, such as catalytic reaction and chemosensors (Ding et al., 2017; To and Chan, 2017; Zhang et al., 2017). Covalent organic frameworks (COFs) are crystalline porous organic polymers linked by covalent bonds with porous and ordered structures, attracting considerable attention due to their porosity, stability, and versatility. The chemical and structural tunability makes them have great potential applications in catalysis, separation, gas storage (Geng et al., 2020; Lee et al., 2020; Li et al., 2020). Moreover, many examples of COFs-based metalloporphyrin building blocks have been fabricated to apply in different catalytic fields (Singh et al., 2018; Cheung et al., 2019; Hao et al., 2019; Gong et al., 2020).

**FIGURE 4:**
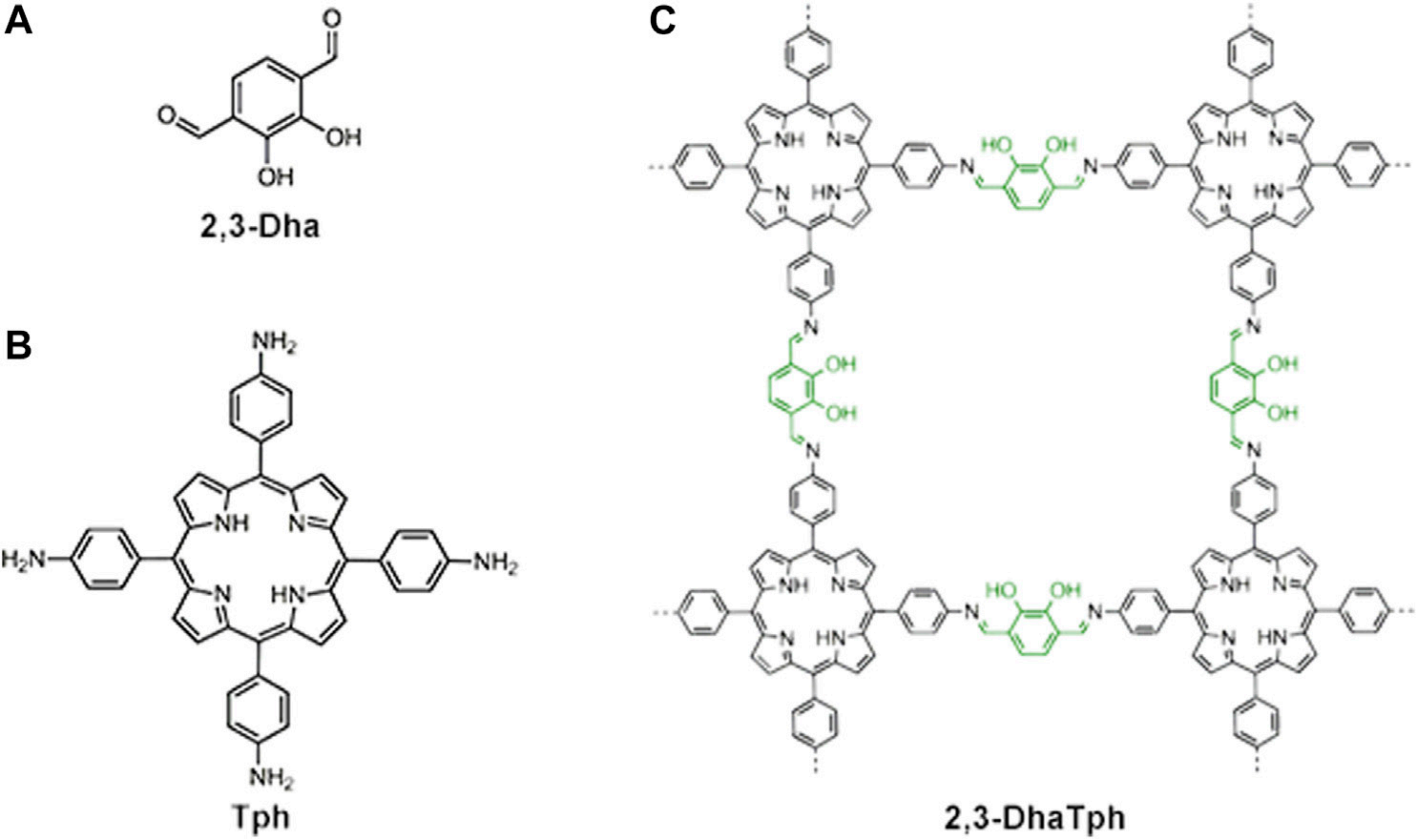
The chemical structures of 2,3-Dha **(A)**, Tph **(B)**, and 2,3-DhaTph **(C)**. The hydroxy groups (green) act as acidic sites, whereas porphyrin and imine bonds (black) act as basic sites for an acid-base catalyzed cascade reaction. Adapted from Shinde et al. (2015) with permission. Copyright 2015 the Royal Society of Chemistry.

One important reaction for the fabrication of COFs is Shiff-base condensation between aldehyde and amine. It is an important strategy to introduce different amino groups and aldehyde groups to porphyrin building blocks, design linkers of porphyrin building blocks, and change metallic ions to construct COFs based on metalloporphyrin. Some examples have achieved recyclability with high catalytic activity. Banerjee group reported a COF (2,3-DhaTph) incorporating bifunctional (acid/base) catalytic sites based on porphyrin (Shinde et al., 2015). By reversible Schiff-base reaction using 2,3-dihydroxyterephthalaldehyde (2,3-Dha, Figure 4A) and a 5,10,15,20-tetrakis (4-amino phenyl)-21H, 23H-porphine unit (Tph, Figure 4B), The COF 2,3-DhaTph (Figure 4C) was synthesized. This COF has high thermal stability up to 300°C and could keep well aqueous stability for more than 7 days. The COF with weak acidic and basic sites could most significantly catalyze the cascade reaction with high product yield (∼90%) and possess recyclability over five cycles. According to similar strategies, Dai’s group successfully introduced Fe^2+^ to COF-366 and detected the oxidation using the Fe-COF as peroxidase Mimics (Wang et al., 2018). The Fe-COF can catalyze the H_2_O_2_ oxidation to show that Fe-COF has an inner peroxidase-like catalytic activity. Additionally, the kinetic studies demonstrated that the Fe-COF structure has a higher affinity toward the substrates than the natural enzyme, horseradish peroxidase. Furthermore, the Fe-COF could be applied in a colorimetric sensor for the sensitive detection of H_2_O_2_ and measure glucose. As a peroxidase mimic, the Fe-COF exhibits the advantages of easy preparation and ultrahigh catalytic efficiency. In addition, the covalent bonds linking macrocycle monomers largely enhanced the stability of catalysts, allowing more practical applications in industry than those of macrocycle systems constructed by weak interactions.

## Metal−Organic Framework Materials as Bioinspired Catalysts

**FIGURE 5:**
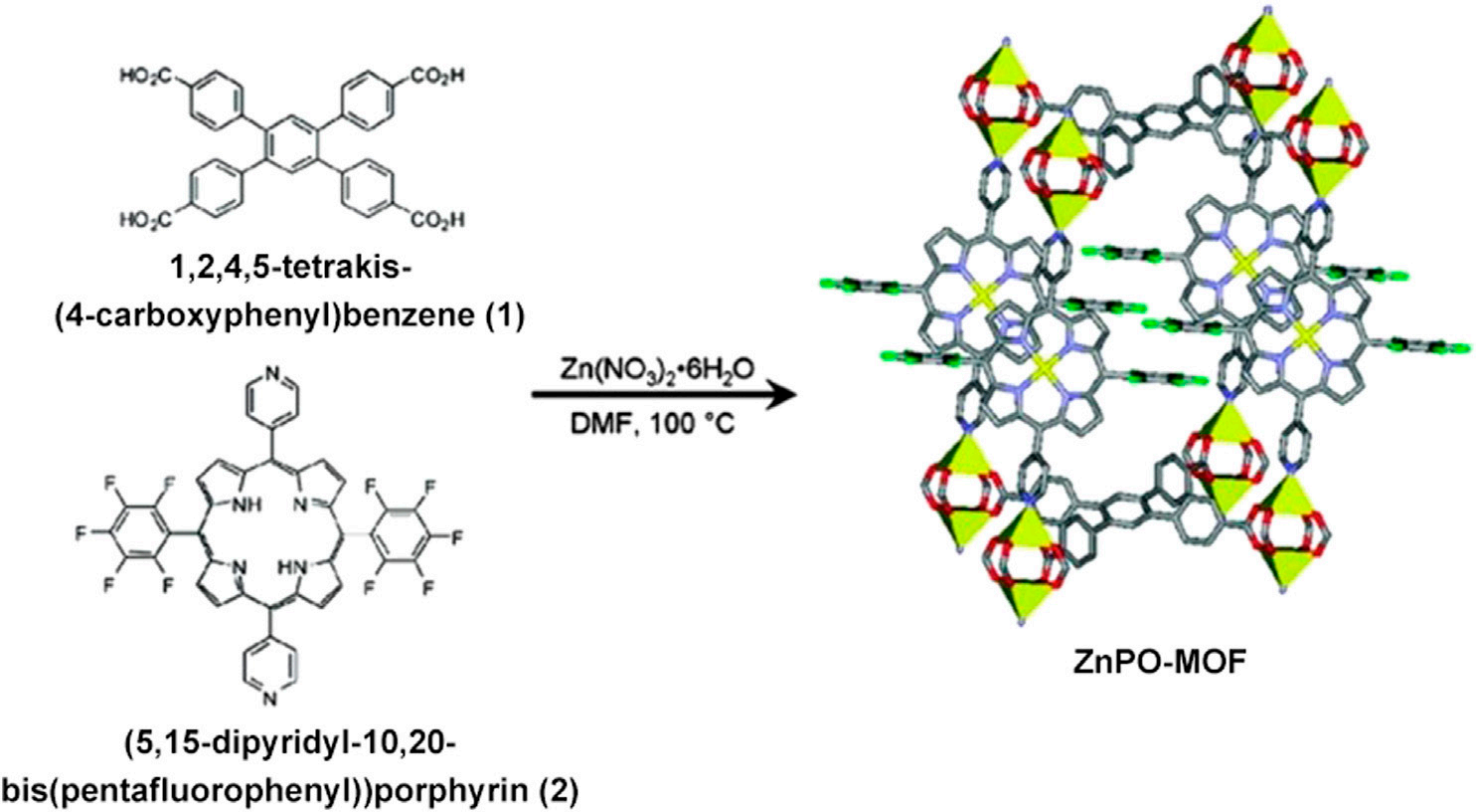
The synthesis of ZnPO-MOF. The MOF (right) has multiple features for catalysis, such as large pores, permanent microporosity, and fully reactant-accessible active sites. Reproduced from Shultz et al. (2009) with permission. Copyright 2009 American Chemical Society.

Metal-organic frameworks (MOFs) are a class of porous crystalline materials based on the coordination of metal ions/clusters and organic linkers. Unlike other materials, a significant MOF feature is that their structure can be facilely designed, functionalized, and offer a high surface area. They have attracted considerable attention in these years and have many potential applications in storage, catalysis, and separation (Cui et al., 2016; Yang et al., 2017; Wei et al., 2020). In recent years, multilevel MOFs using porphyrin as building blocks have been developed to mimic catalyst. As a pioneering example of using metalloporphyrin as a building block to design MOF, in 2009 Nguyen’s group reported a mixed-ligand strategy that combines 1,2,4,5-tetrakis-(4-carboxyphenyl)benzene 1) (5,15-dipyridyl-10,20-bis[pentafluorophenyl])porphyrin 2) and Zn(NO_3_)_2_·6H_2_O under solvothermal conditions (Figure 5) obtained purple block crystals of Zn-MOF with large pores (surface area of ∼500 m^2^/g), and further studies demonstrated that the Zn-MOF is available for a catalytic acyl-transfer reaction between a N-acetylimidazole and 3-pyridinemethanol with huge rate enhancement (Shultz et al., 2009). For the catalytic reaction, Zn serves as a catalytic site, and the size of the cavity plays an essential role in enhancing the reaction rate. Based on this work, Nguyen group introduced different metallic ion (Al^3+^, Zn^2+^, Pd^2+^, Fe^3+^, and Mn^3+^) to coordinate with porphyrin in this system to obtain different COFs materials, which can serve as effective catalysts for the oxidation of alkenes and alkanes (Farha et al., 2011). The work offered a strategy to incorporate macrocyclic metalloporphyrin with catalytic property into nanomaterials to develop heterogeneous catalysts. So, many MOFs based on metalloporphyrin with catalytic activity have been developed, which were designed by changing substitutes in porphyrin, metallic ion coordinating with porphyrin, metal nodes or organic linkers (Feng et al., 2013; Beyzavi et al., 2015; Liu et al., 2015; Lin et al., 2016; Jiang et al., 2017; Liang et al., 2018; Pereira et al., 2019). For instance, Zhou’s group prepared a series of isostructural zirconium-based MOFs, which differ in porphyrin unit functionalized by ethyl, bromo, chloro, and fluoro groups to study the electron effect of substitutes on the catalytic activity of MOFs. These structures showed excellent structural tunability and outstanding chemical stability. The oxidation of 3-methylpentane to corresponding alcohols and ketones was utilized as a model reaction to study the catalytic activity and selectivity. Study results demonstrated that the Br-containing MOF catalyst showed higher catalytic efficiency and a remarkable 99% selectivity of the tertiary alcohol over the five other possible byproducts (Huang et al., 2017).

The disadvantages of the traditional homogeneous catalysts, including difficult recyclability and easy deactivation, limit industry application. Some examples of porphyrin-MOFs have realized high activity and recyclability of the heterogeneous catalyst compared to the homogeneous analog catalysts (Feng et al., 2013). Zhou group designed and prepared a perfluorophenylene functionalized metalloporphyrins MOF (PCN-624) which was constructed by 12-connected [Ni_8_(OH)_4_(H_2_O)_2_Pz_12_] (Pz = pyrazolide) nodes and porphyrin derivative linker. This MOF exhibits excellent stability under different conditions, such as organic solvents, strong acid, and aqueous-based solutions. Attractively, the MOF can be used as an efficient heterogeneous catalyst for the selective synthesis of fullerene−anthracene bisadduct and recycled over five times without significant loss of its catalytic efficiency (Huang et al., 2018). Additionally, many metalloporphyrin-MOFs have also been developed to use as an electrochemical catalyst and photochemical catalyst, which were designed through similar strategies (Kornienko et al., 2015; Jin et al., 2020). These porous crystalline materials could not only provide well-defined molecular recognition sites (Tashiro and Shionoya, 2020), but also metal ions with catalytic activities, combining the keys to making natural enzymes highly efficient.

## Outlook

From the single-molecular level to materials, macrocycles have been developed for various functional building blocks. The unique cavity-like structures of the macrocyclic compounds could promote the catalytic reactions in a bioinspired way. Not only novel macrocycles modified with catalytic functional groups expanded the application scope of macrocycle catalysis, but the supramolecular assembly of macrocycles brought dynamic control and processive catalysis into this research area. In addition, the covalent crosslinking further endowed catalytic porphyrin macrocycles with stability and clustering effects. The combination of macrocycles and catalysis is going to prove its economic value and its academic potential.

However, there are still many challenges ahead in this field. For instance, macrocycle catalysts’ substrate-recognition ability is generally weak compared to natural enzymes, which is the main reason for the relatively low catalytic activity. Also, the catalytic selectivity of macrocycle catalysts is limited by the simplicity of the substrate-binding cavity. Nevertheless, these macrocycles provide novel scaffolds to construct advanced catalysts for synthetic routes. We believe that elaborate structures and practical activities are going to emerge.
